# Initial ribociclib plus endocrine therapy for HR+/HER2− advanced breast cancer in pre‐ and postmenopausal Chinese women: Primary results from a phase 2 randomized study

**DOI:** 10.1002/cam4.7408

**Published:** 2024-08-13

**Authors:** Zhimin Shao, Zhongsheng Tong, Qiang Liu, Wei Li, Li Cai, Kunwei Shen, Huiping Li, Chuan Wang, Jin Yang, Zhenchuan Song, Shui Wang, Ting Luo, Wenhe Zhao, Haibo Wang, Yueyin Pan, Jianyun Nie, Xiaohua Zeng, Yanqing Bai, Wendy Chiang, Valeria Guarnaccia, Yu Bi, Binghe Xu

**Affiliations:** ^1^ Fudan University Shanghai Cancer Center Shanghai China; ^2^ Tianjin Medical University Cancer Institute and Hospital Tianjin China; ^3^ Sun Yat‐Sen Memorial Hospital Sun Yat‐Sen University Guangzhou China; ^4^ The First Bethune Hospital of Jilin University Changchun China; ^5^ Harbin Medical University Cancer Hospital Harbin China; ^6^ RuiJin Hospital Shanghai Jiao Tong University School of Medicine Shanghai China; ^7^ Department of Breast Oncology, Key Laboratory of Carcinogenesis and Translational Research (Ministry of Education/Beijing) Peking University Cancer Hospital & Institute Beijing China; ^8^ Affiliated Union Hospital of Fujian Medical University Fuzhou China; ^9^ The 1st Affiliated Hospital of Xi'an Jiaotong University Xi'An China; ^10^ Fourth Hospital of Hebei Medical University Shijiazhuang China; ^11^ Jiang Su Province RenMin Hospital Nanjing China; ^12^ West China Hospital Sichuan University Chengdu China; ^13^ Sir Run Run Shaw Hospital/Zhejing University School of Medicine Hangzhou China; ^14^ The Affiliated Hospital of Qingdao University Qingdao China; ^15^ Anhui Provincial Hospital Hefei China; ^16^ Yunnan Provincial Cancer Hospital Kunming China; ^17^ Chongqing Cancer Hospital Chongqing China; ^18^ Novartis Global Drug Development Beijing China; ^19^ Novartis Pharmaceuticals Corporation East Hanover New Jersey USA; ^20^ Novartis Pharmaceuticals Corporation Basel Switzerland; ^21^ National Cancer Center/National Clinical Research Center for Cancer/Cancer Hospital, Chinese Academy of Medical Sciences & Peking Union Medical College Beijing China

## Abstract

**Background:**

The MONALEESA‐7 and ‐2 phase 3 randomized trials demonstrated a statistically significant progression‐free survival (PFS) and overall survival (OS) benefit with initial ribociclib + endocrine therapy (ET) versus placebo + ET in pre‐ and postmenopausal patients with hormone receptor–positive (HR+)/human epidermal growth factor receptor 2–negative (HER2−) advanced breast cancer (ABC), respectively. Similar trends were observed in Asian subgroup analyses. This phase 2 bridging study of initial ET + ribociclib enrolled pre‐ and postmenopausal patients with HR+/HER2– ABC from China and was conducted to demonstrate consistency of PFS results in a Chinese population relative to the global MONALEESA‐7 and ‐2 studies.

**Methods:**

Patients were randomized (1:1) to ET (nonsteroidal aromatase inhibitor + goserelin for premenopausal patients; letrozole for postmenopausal patients) + either ribociclib or placebo. The primary endpoint was investigator‐assessed PFS.

**Results:**

As of April 25, 2022, the median follow‐up was 34.7 months in both cohorts. In the premenopausal cohort, median PFS was 27.6 months in the ribociclib arm (*n* = 79) versus 14.7 months in the placebo arm (*n* = 77) (hazard ratio 0.67 [95% CI: 0.45, 1.01]). In the postmenopausal cohort, median PFS was not reached in the ribociclib arm versus 18.5 months in the placebo arm (*n* = 77 in each arm) (hazard ratio 0.40 [95% CI: 0.26, 0.62]). Data also suggested improvements in secondary efficacy endpoints, although OS data were not mature. The safety profile in this population was consistent with that in global studies.

**Conclusions:**

These data demonstrate a favorable benefit–risk profile for ribociclib + ET in Chinese patients.

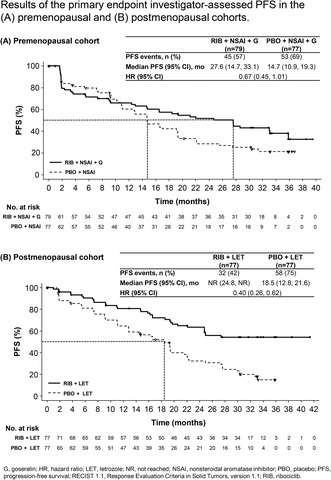

## 1 INTRODUCTION

Breast cancer is the most frequently diagnosed cancer globally and a leading cause of cancer‐related death in women, with notable geographic variations in incidence and mortality.^1^, ^2^ In China, the incidence of female breast cancer has steadily increased over the last 20–30 years,^2^, ^3^ and reductions in mortality seen in many other countries have not been observed.^2^, ^3^, ^4^ Of note, peak breast cancer incidence in women is approximately 10 years earlier in China than in Western countries,^5^ although it may be slowly increasing in China.^2^

Hormone receptor–positive (HR+)/human epidermal growth factor receptor 2–negative (HER2−) breast cancer is the most common breast cancer subtype,^6^, ^7^, ^8^ with endocrine therapy (ET) playing a central role as the therapeutic backbone.^9^ In the advanced breast cancer (ABC) setting, the combination of ET with cyclin‐dependent kinase 4 and 6 (CDK4/6) inhibitors can combat endocrine resistance mechanisms associated with ET, and, as such, represents a contemporary therapeutic breakthrough.^10^ In China, several CDK4/6 inhibitors, including abemaciclib, palbociclib, and dalpiciclib, were approved in combination with ET for post‐ or premenopausal HR+/HER2− ABC and were included in the 2022 treatment recommendations for Chinese patients.^9^ Subsequently, in early 2023, the CDK4/6 inhibitor ribociclib was also approved in China for HR+/HER2− ABC.^11^

Ribociclib + ET demonstrated statistically significant progression‐free survival (PFS) and overall survival (OS) benefits versus ET as initial treatment for patients with HR+/HER2− ABC in 3 randomized, phase 3 placebo‐controlled global studies^12^, ^13^, ^14^: MONALEESA‐3 evaluated ribociclib/placebo with fulvestrant as first‐ or second‐line therapy in postmenopausal patients; MONALEESA‐7 evaluated first‐line ribociclib/placebo with goserelin and either tamoxifen or a nonsteroidal aromatase inhibitor (NSAI) in premenopausal patients; and MONALEESA‐2 evaluated first‐line ribociclib/placebo with letrozole in postmenopausal patients. As first‐line studies, MONALEESA‐7 and ‐2 enrolled patients from different regions, with patients of Asian race representing approximately 30% of patients (*n* = 198) in MONALEESA‐7^15^ and approximately 8% (*n* = 51) in MONALEESA‐2.^16^

Exploratory subgroup analyses from MONALEESA‐7 suggested that Asian patients derive notable clinical benefit from ribociclib + ET (Asian subgroup, PFS hazard ratio [HR] 0.40 [95% CI: 0.26, 0.63] and OS HR 0.40 [95% CI: 0.22, 0.72]; intention‐to‐treat [ITT] population, PFS HR 0.55 [95% CI: 0.44, 0.69] and OS HR 0.71 [95% CI: 0.54, 0.95]),^13^, ^15^ as did those from MONALEESA‐2 (Asian subgroup, PFS HR 0.39 [95% CI: 0.17, 0.91] and OS HR 0.80 [95% CI: 0.42, 1.54]; ITT population, PFS HR 0.56 [95% CI: 0.43, 0.72] and OS HR 0.76 [95% CI: 0.63, 0.93]).^14^, ^16^ Collectively, MONALEESA‐7 and ‐2 included patients from Hong Kong, Korea, Malaysia, Singapore, Taiwan, and Thailand but not mainland China.^15^, ^16^ Prior to regulatory approval in China, a phase 2 study of ribociclib + ET as first‐line treatment in pre‐ and postmenopausal Chinese women with HR+/HER2− ABC was conducted to bridge results from the MONALEESA studies to a Chinese population. Here, we report the primary PFS analysis and key secondary endpoints from the pre‐ and postmenopausal cohorts of this study.

## 2 METHODS

### 2.1 Study design and patients

This phase 2, randomized double‐blind, placebo‐controlled study (ClinicalTrials.gov ID, NCT03671330) evaluated ribociclib + ET in women with HR+/HER2− ABC. Three cohorts of patients were enrolled in mainland China study sites: pharmacokinetics, premenopausal, and postmenopausal cohorts. Results from the pre‐ and postmenopausal cohorts are reported here.

Eligible patients ≥18 years old had locoregionally recurrent or metastatic HR+/HER2− breast cancer (based on most recently analyzed biopsy). Patients must have had Eastern Cooperative Oncology Group performance status (ECOG PS) of 0/1 and adequate bone marrow and organ function. Patients must have had either measurable disease per Response Evaluation Criteria in Solid Tumors (RECIST) 1.1 criteria or ≥1 predominantly lytic bone lesion; the latter were eligible if disease progression was seen following irradiation of this lesion.

Patients must have been eligible for ET and not received prior hormonal therapy for ABC. Patients who received ≤ 14 days of letrozole or anastrozole (± goserelin) or ≤ 28 days of goserelin for ABC prior to randomization were eligible but must have continued the same hormonal agent plus goserelin, if premenopausal, during the study. Patients could have had ABC that was either newly diagnosed, treatment naive with progression > 12 months following anastrozole‐ or letrozole‐based therapy, or previously treated with (neo)adjuvant therapy, provided therapy was stopped ≥ 5 half‐lives or 7 days, whichever was longer, before randomization. If letrozole, anastrozole, or exemestane was the last (neo)adjuvant therapy, the last dose must have been ≥ 12 months before randomization; for other (neo)adjuvant endocrine agents, no limitations were placed on recurrence timing.

In the premenopausal cohort, patients must have been < 60 years old at the time of informed consent. Premenopausal status was defined by one of the following: a menstrual period in the last 12 months; if on tamoxifen or toremifene in the past 14 days, plasma estradiol ≥ 10 pg/mL and follicle‐stimulating hormone (FSH) ≤  40 IU/L or in the premenopausal range (local laboratory); or in the case of therapy‐induced amenorrhea, plasma estradiol ≥ 10 pg/mL and/or FSH ≤ 40 IU/L or in the premenopausal range (local laboratory). Patients who received ≤  1 line of chemotherapy for ABC were eligible if treatment was discontinued 28 days before randomization; if the chemotherapy regimen was discontinued for reasons other than disease progression and was given for < 21 days, the regimen was not counted as a prior line.

Postmenopausal status was defined by one of the following: prior bilateral oophorectomy; age ≥ 60 years; age < 60 years with amenorrhea for ≥ 12 months (in the absence of chemotherapy, tamoxifen, toremifene, or ovarian suppression); and FSH and estradiol in the postmenopausal range (local normal range). For women with therapy‐induced amenorrhea, serial measurements of FSH and/or estradiol were needed to ensure postmenopausal status.

Patients were ineligible if they had inflammatory breast cancer, prior therapy with a CDK4/6 inhibitor, symptomatic visceral disease, disease burden rendering them ineligible for ET at the investigator's discretion, or central nervous system metastases. Patients with clinically relevant, uncontrolled heart disease and/or cardiac repolarization abnormalities were also ineligible.

### 2.2 Randomization and blinding

A double‐blind, placebo‐controlled design was used in both the pre‐ and postmenopausal cohorts, with patients randomized 1:1 to each treatment arm. Randomization was stratified by the presence of lung and/or liver metastases (yes vs. no) in both cohorts and additionally by receipt of prior chemotherapy for advanced disease (yes vs. no) in the premenopausal cohort only. The patients, investigators, study team, and those involved in study conduct, including local or sponsor‐designated radiologists, remained blinded to the treatment identity from randomization until database lock. An independent, unblinded team from the sponsor, not involved in the study conduct or study drug development program, performed 2 interim analyses of the premenopausal cohort, which did not change the study's blinding mechanism, for the sole purpose of sharing results with the health authority. Unblinding of the treatment assignment could occur in the case of medical emergencies, for regulatory reporting purposes, and at conclusion of study. Unblinding for safety was communicated only to an independent statistical group external to the sponsor for reporting to the data monitoring committee (DMC).

### 2.3 Study treatment, assessments, and conduct

During each 28‐day cycle, patients in the premenopausal cohort received letrozole 2.5 mg or anastrozole 1 mg (orally, once daily) plus goserelin 3.6 mg (as a subcutaneous implant on day 1) as well as ribociclib 600 mg (experimental arm; orally, once daily on days 1–21) or matched placebo (comparator arm). Patients in the postmenopausal cohort received letrozole with either ribociclib or matched placebo using the same dosing regimens based on a 28‐day cycle. Dose modifications of letrozole, anastrozole, or goserelin were not permitted, but dose reductions of ribociclib/placebo were permitted for tolerability per the investigator's judgment (2 dose reductions maximum, from 600 to 400 to 200 mg/day). Ribociclib dose could not be re‐escalated once decreased.

Patients were treated until disease progression, unacceptable toxicity, withdrawal of consent, loss to follow‐up, or study termination. For patients who discontinued treatment for reasons other than disease progression (including initiation of another antineoplastic therapy), tumor assessments were performed every 8 weeks for 18 months, followed by every 12 weeks until 36 months, and thereafter as clinically indicated until disease progression, death, withdrawal of consent, loss to follow‐up, or patient/guardian decision. Patients who discontinued treatment were followed up for safety for ≥ 30 days unless they died, became lost to follow‐up, or withdrew consent. All patients were followed up for survival at least every 12 weeks unless consent was withdrawn. The study was considered completed when all patients had discontinued from the study, died, or became eligible to transfer to another clinical study that provided ribociclib to the patient population, whichever occurred first.

Adverse events (AEs) were recorded per Common Terminology Criteria for Adverse Events version 4.03. Additional safety assessments, including physical examination, ECOG PS, echocardiogram, and laboratory tests, were also performed. The DMC reviewed safety data approximately every 6 months in an unblinded manner.

A steering committee that included study investigators and study team members from the sponsor provided study oversight. They were consulted regarding protocol amendments and provided recommendations on publication of study results. The steering committee did not have access to unblinded trial data but did review DMC recommendations and communicated responses to other investigators.

Permitted changes to study conduct due to the COVID‐19 pandemic included the possibility for additional study drug to be sent home when considered safe or special arrangements for study treatment via courier or mail service. Onsite monitoring visits were replaced with remote visits, and monitors were directed to consider aspects of site trial management susceptible to the pandemic challenges. The investigators could use local assessments or laboratories when appropriate.

### 2.4 Study endpoints

The primary endpoint in both the pre‐ and postmenopausal cohorts was PFS, locally assessed according to RECIST 1.1, defined as the time from randomization to the first documented progression or death due to any cause. PFS was also assessed in subgroups defined by demographic and clinical characteristics to evaluate the consistency of the study results.

Secondary endpoints in the pre‐ and postmenopausal cohorts were OS, locally assessed RECIST 1.1 overall response rate (ORR), clinical benefit rate (CBR), time to response (TTR), duration of response (DOR), and time to deterioration of ECOG PS. Safety and tolerability were also secondary objectives.

### 2.5 Statistical analysis

The study pursued an estimation strategy for each cohort rather than formal hypothesis testing to demonstrate the consistency of PFS with ribociclib + ET versus placebo + ET in Chinese populations compared with populations in previous global studies (MONALEESA‐7 for the premenopausal cohort and MONALEESA‐2 for the postmenopausal cohort). In each of the pre‐ and postmenopausal cohorts, 150 patients were planned to be randomized in a 1:1 ratio to observe 100 PFS events at the time of primary analysis, approximately 23 months after the first patient was randomized. A total of 3 interim analyses were performed, and only the primary analysis of PFS is included in this report.

Efficacy analyses were evaluated in the full analysis set (ITT population) in each respective cohort, which comprised all randomized patients assigned to study treatment. The safety population included all patients who received ≥ 1 dose of any component of study treatment.

The Kaplan–Meier method was used for estimation of the PFS distribution. A Cox regression model stratified by the randomization stratification factors was used to estimate the PFS HR and 95% CI. Patients whose disease had not progressed or were not known to have died were censored at the time of the last adequate tumor assessment prior to the cutoff. If a PFS event was documented after ≥ 2 missing or inadequate tumor assessments, censoring was done at the date of the last adequate tumor assessment. If a PFS event was observed after a single missing or inadequate tumor assessment, the actual date of the PFS event was used. For analysis of response, ORR was presented with Wald 95% CIs. TTR and DOR were also estimated using Kaplan–Meier methods. For OS, the number of events in each arm was reported. For PFS and OS, follow‐up was defined from randomization to the date of event or censoring (as described above for the PFS analysis) plus 1 day, expressed in months.

Safety summaries included AEs occurring on or after the first date of study treatment through ≤  30 days after the date of last study treatment administration.

SAS 9.4 software was used to perform data analyses and generate tables and listings.

## 3 RESULTS

### 3.1 Patients

The study was initiated on August 29, 2018, the time of the first patient's first visit. The primary analysis data cutoff was April 25, 2022. Median follow‐up from randomization to data cutoff was 34.7 months (range, 31.0–43.5). Patients were enrolled across 25 study sites in mainland China (Table S1): 79 and 77 patients in the ribociclib and placebo arms of the premenopausal cohort, respectively, and 77 in each arm of the postmenopausal cohort. Baseline characteristics were generally balanced between arms in each cohort (Table 1), with a few exceptions in the postmenopausal cohort (e.g., the placebo arm had higher percentages of stage IV disease at initial diagnosis and de novo metastatic disease and had a shorter median duration since initial primary diagnosis).

**TABLE 1:** Baseline characteristics.

Baseline characteristic	Premenopausal		Postmenopausal	
	RIB + NSAI + G (*n* = 79)	PBO + NSAI + G (*n* = 77)	RIB + LET (*n* = 77)	PBO + LET (*n* = 77)
Female, *n* (%)	79 (100)	77 (100)	77 (100)	77 (100)
Age, median (range), y	44.0 (30–59)	46.0 (22–57)	59.0 (37–78)	60.0 (36–80)
Asian race, *n* (%)	79 (100)	77 (100)	77 (100)	77 (100)
ECOG PS, *n* (%)				
0	43 (54.4)	46 (59.7)	48 (62.3)	43 (55.8)
1	36 (45.6)	31 (40.3)	29 (37.7)	34 (44.2)
Stage at initial diagnosis				
I	5 (6.3)	6 (7.8)	10 (13.0)	3 (3.9)
II	26 (32.9)	36 (46.8)	26 (33.8)	18 (23.4)
III	17 (21.5)	10 (13.0)	17 (22.1)	17 (22.1)
IV	26 (32.9)	20 (26.0)	14 (18.2)	27 (35.1)
Unknown	5 (6.3)	5 (6.5)	10 (13.0)	12 (15.6)
Stage IV at study entry, *n* (%)^a^	79 (100)	77 (100)	76 (98.7)	75 (97.4)
Hormone receptor–positive status, *n* (%)				
ER	78 (98.7)	77 (100)	76 (98.7)	77 (100)
PR	64 (81.0)	70 (90.9)	64 (83.1)	66 (85.7)
ER and/or PR	79 (100)	77 (100)	77 (100)	77 (100)
HER2‐negative status, *n* (%)	79 (100)	77 (100)	77 (100)	77 (100)
No. of metastatic sites, *n* (%)				
1	15 (19.0)	19 (24.7)	17 (22.1)	19 (24.7)
2	27 (34.2)	21 (27.3)	20 (26.0)	20 (26.0)
≥ 3	37 (46.8)	37 (48.1)	40 (51.9)	38 (49.4)
Site(s) of metastasis, *n* (%)^b^				
Soft tissue	15 (19.0)	10 (13.0)	23 (29.9)	21 (27.3)
Bone	56 (70.9)	48 (62.3)	43 (55.8)	43 (55.8)
Bone only	8 (10.1)	9 (11.7)	3 (3.9)	7 (9.1)
Breast	7 (8.9)	3 (3.9)	2 (2.6)	3 (3.9)
Viscera	50 (63.3)	49 (63.6)	45 (58.4)	48 (62.3)
Lung	33 (41.8)	38 (49.4)	43 (55.8)	41 (53.2)
Liver	24 (30.4)	21 (27.3)	10 (13.0)	9 (11.7)
Lung or liver	47 (59.5)	49 (63.6)	44 (57.1)	45 (58.4)
Other^c^	11 (13.9)	13 (16.9)	17 (22.1)	15 (19.5)
Skin	2 (2.5)	2 (2.6)	3 (3.9)	5 (6.5)
Lymph node	44 (55.7)	45 (58.4)	53 (68.8)	54 (70.1)
Time from initial diagnosis (primary tumor site) to study entry, median (range), mo	35.0 (0.5–119.9)	35.6 (0.3–195.7)	75.3 (0.3–300.9)	43.7 (0.3–265.8)
DFI, *n* (%)^b^				
De novo	10 (12.7)	16 (20.8)	15 (19.5)	28 (36.4)
Non‐de novo	69 (87.3)	61 (79.2)	62 (80.5)	49 (63.6)
≤ 12 months	18 (22.8)	7 (9.1)	2 (2.6)	4 (5.2)
> 12 and ≤ 24 months	5 (6.3)	7 (9.1)	0	1 (1.3)
> 24 months	46 (58.2)	47 (61.0)	60 (77.9)	44 (57.1)
Prior antineoplastic therapy, *n* (%)^c^				
No	11 (13.9)	16 (20.8)	21 (27.3)	32 (41.6)
Yes	68 (86.1)	61 (79.2)	56 (72.7)	45 (58.4)
Prior regimens, *n* (%)^d^				
1	67 (84.8)	61 (79.2)	56 (72.7)	45 (58.4)
≥ 2	6 (7.6)	17 (22.1)	1 (1.3)	2 (2.6)
Most recent type of therapy, *n* (%)^e^				
Chemotherapy	19 (24.1)	24 (31.2)	26 (33.8)	13 (16.9)
Hormonal therapy	30 (38.0)	25 (32.5)	21 (27.3)	17 (22.1)
Targeted therapy	0	1 (1.3)	0	0
Not available	1 (1.3)	2 (2.6)	0	0
Most recent treatment setting, *n* (%)^e^				
Adjuvant	32 (40.5)	31 (40.3)	46 (59.7)	29 (37.7)
Metastatic	17 (21.5)	17 (22.1)	0	0
Neoadjuvant	0	2 (2.6)	0	1 (1.3)
Other	0	1 (1.3)	0	0
Prior ET (neoadjuvant or adjuvant setting), *n* (%)^e^				
None	39 (49.4)	35 (45.5)	47 (61.0)	52 (67.5)
Yes, progression on or within 12 mo after end of ET	32 (40.5)	31 (40.3)	5 (6.5)	7 (9.1)
Yes, progression > 12 mo after end of ET	8 (10.1)	11 (14.3)	25 (32.5)	18 (23.4)

Abbreviations: DFI, disease‐free interval; ECOG PS, Eastern Cooperative Oncology Group performance status; ER, estrogen receptor; ET, endocrine therapy; G, goserelin; HER2, human epidermal growth factor receptor 2; LET, letrozole; NSAI, nonsteroidal aromatase inhibitor; PBO, placebo; PR, progesterone receptor; RIB, ribociclib.

^a^ All patients in the premenopausal cohort had stage IV disease at study entry; in the postmenopausal cohort, 1 patient (1.3%) in the RIB arm and 2 (2.6%) in the PBO arm had stage III disease at study entry.

^b^ One patient (1.3%) in the PBO arm of the postmenopausal cohort had pancreas metastases. One patient in the PBO arm of the postmenopausal cohort had metastatic site information that was not available.

^c^ Other refers to any metastatic site other than soft tissue, bone, lung, liver, skin, or lymph node.

^d^ De novo disease category includes patients without initial recurrence or progression or with initial recurrence or progression within 90 days of diagnosis with no prior antineoplastic medication. For non–de novo disease category, DFI refers to the time from initial diagnosis to initial recurrence or progression.

^e^ Data on prior treatment excludes the short period of prior hormonal therapy (NSAI, with or without G) for advanced breast cancer that was permitted by the protocol.

In the premenopausal cohort, median age across treatment arms was 45.0 years (range, 22–59), with 75.0% of patients ≥ 40 years of age. All had baseline stage IV disease, and most (83.3%) had non‐de novo disease. A total of 40.4% of premenopausal patients who received prior (neo)adjuvant ET had progression within 12 months of end of ET. Bone, viscera, and lymph nodes were the most common sites of metastases, present in 66.7%, 63.5%, and 57.1% of premenopausal patients, respectively. In the postmenopausal cohort, the median age was 60 years (range, 36–80), with 71.4% of patients < 65 years of age. In this cohort, 98.7% of patients in the ribociclib arm and 97.4% in the placebo arm had baseline stage IV disease. Most postmenopausal patients (72.1%) had non‐de novo disease (ribociclib, 80.5%; placebo, 63.6%). Lymph nodes, viscera, and bone were the most common metastatic sites, present in 69.5%, 60.4%, and 55.8% of postmenopausal patients, respectively.

In both cohorts, most patients received prior antineoplastic therapy (premenopausal, 82.7%; postmenopausal, 65.6%). The most recent treatment setting for the premenopausal cohort was (neo)adjuvant in 41.7% of patients and metastatic in 21.8%; in the postmenopausal cohort, 49.4% were last treated in the (neo)adjuvant setting. In the premenopausal cohort, the most recent therapy was hormonal therapy in 35.3% of patients and chemotherapy in 27.6%; in the postmenopausal cohort, these classes of therapy were given in 24.7% and 25.3%, respectively.

**FIGURE 1:**
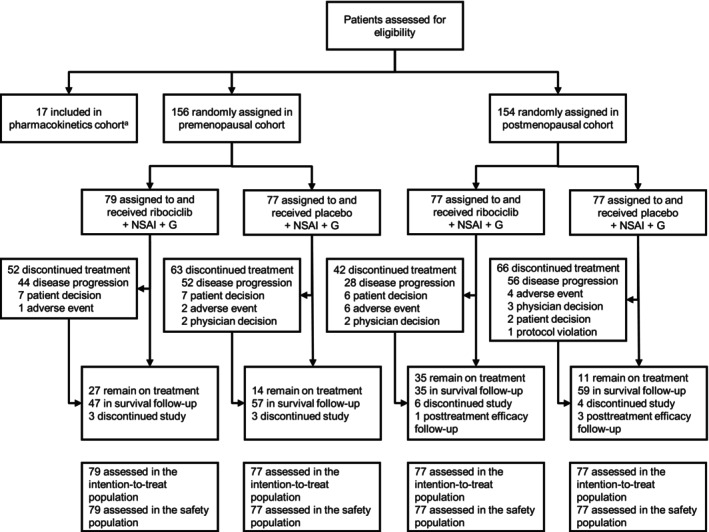
Patient flow diagram. Study cohorts, disposition, and analysis populations are shown. ITT population refers to the full analysis set. G, goserelin; NSAI, nonsteroidal aromatase inhibitor. ^a^The pre‐ and postmenopausal cohorts are reported here.

At data cutoff, 27 (34.2%) and 35 (45.5%) patients in the ribociclib arms versus 14 (18.2%) and 11 (14.3%) in the placebo arms of the pre‐ and postmenopausal cohorts, respectively, remained on treatment (Figure 1). In all arms, most treatment discontinuations (66.7%–84.6%) were due to progressive disease.

### 3.2 PFS (primary endpoint)

**FIGURE 2:**
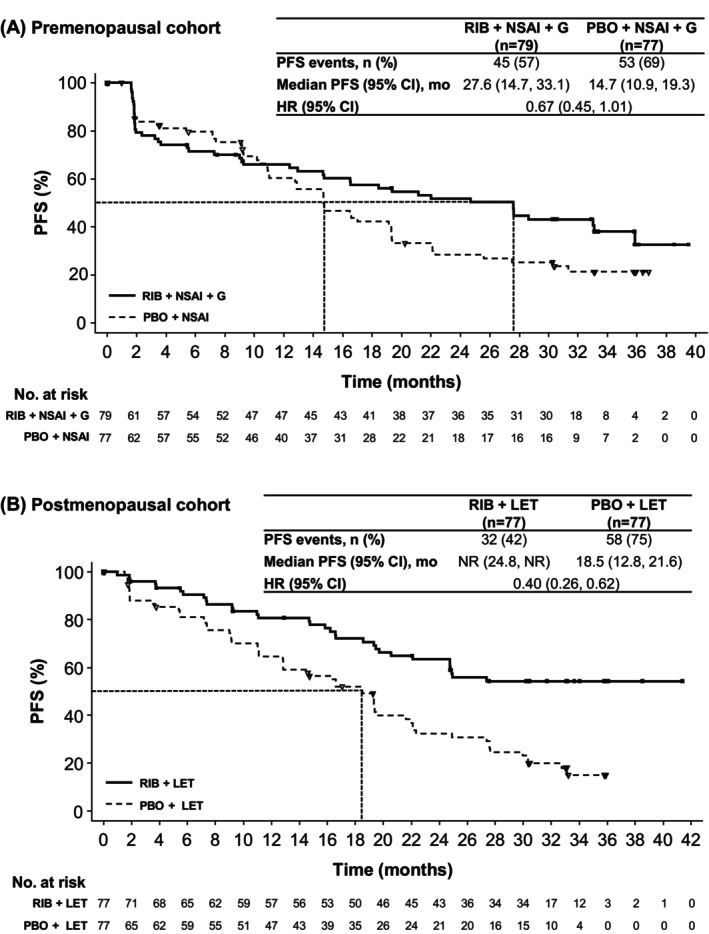
PFS (primary endpoint). PFS was investigator assessed per RECIST 1.1 in the (A) premenopausal and (B) postmenopausal cohorts. G, goserelin; HR, hazard ratio; LET, letrozole; NR, not reached; NSAI, nonsteroidal aromatase inhibitor; PBO, placebo; PFS, progression‐free survival; RECIST 1.1, Response Evaluation Criteria in Solid Tumors, version 1.1; RIB, ribociclib.

**FIGURE 3:**
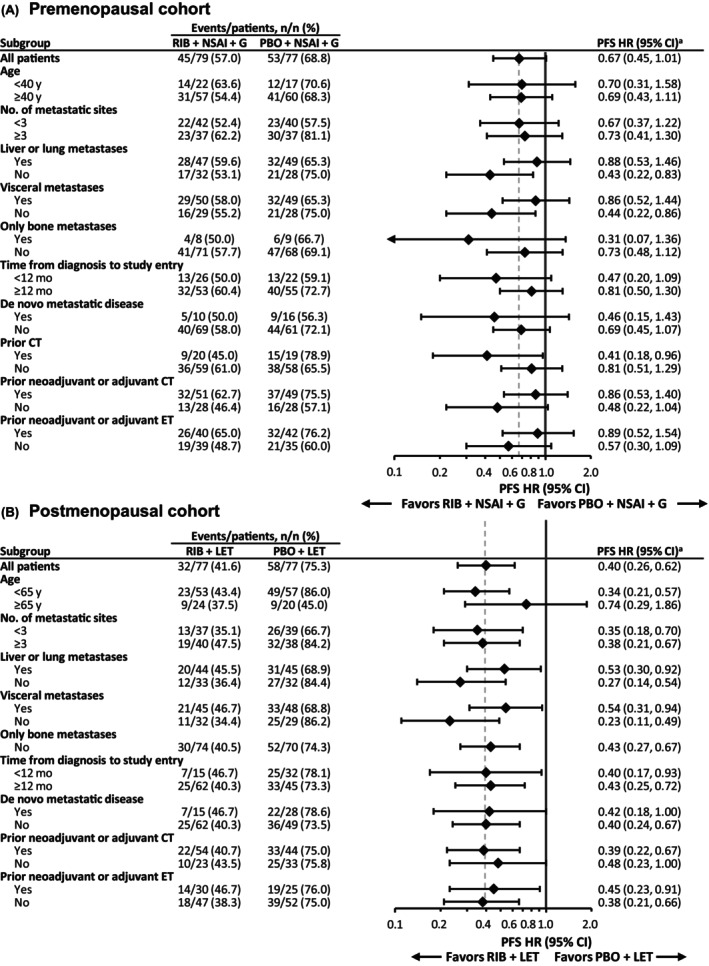
PFS subgroup analysis. Forest plot of investigator‐assessed RECIST 1.1 PFS in the (A) premenopausal and (B) postmenopausal cohorts. CT, chemotherapy; ET, endocrine therapy; G, goserelin; HR, hazard ratio; LET, letrozole; NSAI, nonsteroidal aromatase inhibitor; PBO, placebo; PFS, progression‐free survival; RECIST 1.1, Response Evaluation Criteria in Solid Tumors, version 1.1; RIB, ribociclib. ^a^Stratified HRs based on a Cox proportional hazards model are shown, with the exception of lung/liver involvement and prior chemotherapy in metastatic setting subgroups, for which unstratified HRs are shown.

At the time of primary PFS analysis (Figure 2), median follow‐up for PFS was 14.7 months (range, 0–39.5) in the premenopausal cohort and 19.3 months (0–41.4) in the postmenopausal cohort. Ninety‐eight PFS events (62.8%) had occurred in the premenopausal cohort and 90 (58.4%) in the postmenopausal cohort. In the premenopausal cohort, median PFS was 27.6 months (95% CI: 14.7, 33.1) in the ribociclib arm and 14.7 months (95% CI: 10.9, 19.3) in the placebo arm (HR 0.67 [95% CI: 0.45, 1.01]). The landmark PFS rate at 30 months was 43.1% (95% CI: 31.6%, 54.1%) and 25.2% (95% CI: 15.5%, 36.1%) in the ribociclib and placebo arms, respectively. In the postmenopausal cohort, median PFS was not reached (NR) (95% CI: 24.8 months, NR) in the ribociclib arm and 18.5 months (95% CI: 12.8, 21.6) in the placebo arm (HR 0.40; 95% CI: 0.26, 0.62). The landmark PFS at 30 months was 54.3% (95% CI: 41.9%, 65.2%) and 23.0% (95% CI: 13.8%, 33.5%) in the ribociclib and placebo arms, respectively. PFS results were generally consistent across subgroups (Figure 3).

### 3.3 Key secondary efficacy endpoints

OS data were not mature at the time of analysis (median [range] OS follow‐up, 31.7 months [0.4–42.6] in the premenopausal cohort and 32.0 months [1.1–42.3] in the postmenopausal cohort). Overall, 41 OS events (deaths) were seen in the premenopausal cohort (ribociclib arm, 18 events [22.8%]; placebo arm, 23 events [29.9%]), and 32 were seen in the postmenopausal cohort (ribociclib arm, 12 events [15.6%]; placebo arm, 20 events [26.0%]).

Response data based on patients with measurable disease at baseline are reported in Table 2. In these patients, in the premenopausal cohort, the ORR was 53.6% (*n* = 37/69) (95% CI: 41.2%, 65.7%) in the ribociclib arm and 38.5% (*n* = 25/65) (95% CI: 26.7%, 51.4%) in the placebo arm. The CBR was 69.6% and 72.3%, respectively. In the postmenopausal cohort, the ORR was 60.3% (*n* = 44/73) (95% CI: 48.1%, 71.5%) in the ribociclib arm and 49.3% (*n* = 35/71) (95% CI: 37.2%, 61.4%) in the placebo arm. The CBR was 82.2% and 78.9%, respectively. In premenopausal patients, median TTR was 3.7 months with ribociclib + ET versus 5.5 months with placebo + ET and 3.6 versus 3.7 months in postmenopausal patients. In both pre‐ and postmenopausal cohorts, DOR was numerically longer in the ribociclib arm versus placebo arm (32.2 vs. 17.6 months and NR vs. 19.9 months, respectively) (Table 2).

**TABLE 2:** Secondary efficacy endpoints^a^.

	Premenopausal		Postmenopausal	
	RIB + NSAI + G (*n* = 79)	PBO + NSAI + G (*n* = 77)	RIB + LET (*n* = 77)	PBO + LET (*n* = 77)
Patients with measurable disease at baseline, *n*	69	65	73	71
ORR, *n* (%)^b^	37 (53.6)	25 (38.5)	44 (60.3)	35 (49.3)
95% CI, %	41.2, 65.7	26.7, 51.4	48.1, 71.5	37.2, 61.4
Best overall response, *n* (%)				
CR	4 (5.8)	2 (3.1)	2 (2.7)	1 (1.4)
PR	33 (47.8)	23 (35.4)	42 (57.5)	34 (47.9)
SD	16 (23.2)	26 (40.0)	24 (32.9)	28 (39.4)
PD	14 (20.3)	11 (16.9)	3 (4.1)	7 (9.9)
Unknown	2 (2.9)	3 (4.6)	2 (2.7)	1 (1.4)
CBR, *n* (%)^c^	48 (69.6)	47 (72.3)	60 (82.2)	56 (78.9)
95% CI, %	57.3, 80.1	59.8, 82.7	71.5, 90.2	67.6, 87.7
Time to response, median, mo^d^	3.7	5.5	3.6	3.7
Range, mo	2–15	2–17	2–30	2–22
Duration of response^d^				
Events, *n* (%)	17 (45.9)	16 (64.0)	17 (38.6)	24 (68.6)
Median, mo	32.2	17.6	NR	19.9
95% CI, mo^e^	24.0, NR	9.2, 25.8	23.0, NR	14.0, 24.6

*Note*: The 95% CIs for each variable represent exact binomial confidence intervals.

Abbreviations: CBR, clinical benefit rate; CR, complete response; G, goserelin; LET, letrozole; NR, not reached; NSAI, nonsteroidal aromatase inhibitor; ORR, overall response rate; PBO, placebo; PD, progressive disease; PR, partial response; RIB, ribociclib; SD, stable disease.

^a^ Calculations are based on patients with measurable disease at baseline.

^b^ ORR was defined as the proportion of patients who achieved a confirmed CR or PR.

^c^ CBR was defined as the proportion of patients who achieved a confirmed CR, PR, or SD (non‐CR/non‐PD for ≥ 24 weeks).

^d^ Based on patients with a response.

^e^ The Brookmeyer‐Crowley method was used to calculate 95% CIs for median.

### 3.4 Safety

All patients received ≥ 1 dose of study treatment. In the premenopausal cohort, the median duration of exposure to any component of study treatment was 18.5 months in the ribociclib arm and 13.8 months in the placebo arm; in the postmenopausal cohort, the median duration of exposure was 24.9 and 15.6 months, respectively (Table S2). In general, AEs were the predominant reason for dose reductions, interruptions, and delays (Table S3). Dose reductions due to AEs were more frequent in the ribociclib versus placebo arm (premenopausal, 39.2% vs. 2.6%; postmenopausal, 41.6% vs. 6.5%), with most patients requiring only 1 dose reduction (premenopausal, 32.9% vs. 3.9%; postmenopausal, 29.9% vs. 7.8%). Frequencies of dose interruption due to AEs were 74.7% with ribociclib versus 19.5% with placebo in the premenopausal cohort and 75.3% versus 18.2%, respectively, in the postmenopausal cohort.

All patients in the ribociclib arms and 96.1% in the placebo arms experienced an AE due to any cause (Tables 3 and S4). No on‐treatment deaths were reported in the premenopausal cohort, and 1 per arm (1.3%) was reported in the postmenopausal cohort. The most common all‐grade AEs in the ribociclib versus placebo arms, respectively, were neutropenia (premenopausal, 98.7% vs. 29.9%; postmenopausal, 96.1% vs. 20.8%) and leukopenia (premenopausal, 98.7% vs. 46.8%; postmenopausal, 94.8% vs. 32.5%). In the premenopausal cohort, AEs leading to treatment discontinuation occurred in 5 patients each in the ribociclib versus placebo arms (6.3% vs. 6.5%), the most common being prolonged electrocardiogram (ECG) QT (in 2 vs. 1 patients). In the postmenopausal cohort, AEs leading to treatment discontinuation occurred in 11 patients (14.3%) versus 5 (6.5%), the most common being increased aspartate aminotransferase (AST; in 6 vs. 2 patients) and increased alanine aminotransferase (ALT; in 3 vs. 1 patients). All other AEs leading to discontinuation were low frequency (*n* = 1 each).

**TABLE 3:** Most common AEs regardless of attribution.

	Premenopausal				Postmenopausal			
	RIB + NSAI + G (*n* = 79)		PBO + NSAI + G (*n* = 77)		RIB + LET (*n* = 77)^a^		PBO + LET (*n* = 77)	
Patients with AE, *n* (%)^b^	All grades	Grade 3/4	All grades	Grade 3/4	All grades	Grade 3/4	All grades	Grade 3/4
Any AE	79 (100)	61 (77.2)	74 (96.1)	21 (27.3)	77 (100)	62 (80.5)	74 (96.1)	32 (41.6)
Hematologic AEs (group and PT)								
Neutropenia	78 (98.7)	49 (62.0)	23 (29.9)	3 (3.9)	74 (96.1)	49 (63.6)	16 (20.8)	1 (1.3)
Neutrophil count decreased	70 (88.6)	44 (55.7)	20 (26.0)	3 (3.9)	66 (85.7)	45 (58.4)	14 (18.2)	0
Neutropenia	12 (15.2)	7 (8.9)	4 (5.2)	0	12 (15.6)	6 (7.8)	2 (2.6)	1 (1.3)
Febrile neutropenia	1 (1.3)	1 (1.3)	0	0	0	0	0	0
Leukopenia	78 (98.7)	28 (35.4)	36 (46.8)	1 (1.3)	73 (94.8)	23 (29.9)	25 (32.5)	2 (2.6)
Leukopenia	12 (15.2)	5 (6.3)	7 (9.1)	0	11 (14.3)	3 (3.9)	2 (2.6)	1 (1.3)
White blood cell count decreased	68 (86.1)	23 (29.1)	29 (37.7)	1 (1.3)	62 (80.5)	20 (26.0)	22 (28.6)	1 (1.3)
Lymphocyte count decreased	9 (11.4)	3 (3.8)	1 (1.3)	0	15 (19.5)	2 (2.6)	5 (6.5)	0
Lymphopenia	1 (1.3)	1 (1.3)	0	0	3 (3.9)	0	0	0
Anemia	52 (65.8)	5 (6.3)	13 (16.9)	1 (1.3)	42 (54.5)	3 (3.9)	11 (14.3)	5 (6.5)
Anemia	43 (54.4)	5 (6.3)	12 (15.6)	1 (1.3)	34 (44.2)	2 (2.6)	8 (10.4)	4 (5.2)
Hemoglobin decreased	12 (15.2)	0	1 (1.3)	0	11 (14.3)	1 (1.3)	3 (3.9)	1 (1.3)
Erythropenia	1 (1.3)	0	0	0	0	0	0	0
Red blood cell count decreased	2 (2.5)	0	0	0	0	0	1 (1.3)	0
Thrombocytopenia	31 (39.2)	2 (2.5)	12 (15.6)	1 (1.3)	30 (39.0)	1 (1.3)	14 (18.2)	0
Platelet count decreased	25 (31.6)	2 (2.5)	12 (15.6)	1 (1.3)	27 (35.1)	1 (1.3)	12 (15.6)	0
Thrombocytopenia	6 (7.6)	0	1 (1.3)	0	4 (5.2)	0	3 (3.9)	0
Nonhematologic AEs (PT)								
Electrocardiogram QT prolonged	39 (49.4)	4 (5.1)	4 (5.2)	2 (2.6)	20 (26.0)	1 (1.3)	4 (5.2)	0
Alanine aminotransferase increased	29 (36.7)	2 (2.5)	41 (53.2)	1 (1.3)	30 (39.0)	11 (14.3)	30 (39.0)	5 (6.5)
Aspartate aminotransferase increased	27 (34.2)	2 (2.5)	34 (44.2)	2 (2.6)	33 (42.9)	10 (13.0)	30 (39.0)	3 (3.9)
Gamma‐glutamyltransferase increased	17 (21.5)	8 (10.1)	21 (27.3)	5 (6.5)	17 (22.1)	5 (6.5)	20 (26.0)	6 (7.8)
Blood alkaline phosphatase increased	14 (17.7)	1 (1.3)	10 (13.0)	0	16 (20.8)	0	21 (27.3)	2 (2.6)
Hypertension	2 (2.5)	0	10 (13.0)	2 (2.6)	8 (10.4)	4 (5.2)	10 (13.0)	4 (5.2)

Abbreviations: AE, adverse event; G, goserelin; LET, letrozole; NSAI, nonsteroidal aromatase inhibitor; PBO, placebo; PT, preferred term; RIB, ribociclib.

^a^ Only one grade 5 AE was seen in the ribociclib + letrozole arm, hepatic failure, in a patient who had disease progression in the liver.

^b^ All‐grade events occurring in ≥ 25% and grade ≥ 3 AEs occurring in > 5% of patients in any arm.

AEs of special interest (AESIs; Table S5) were mostly hematologic, as described above. The most common nonhematologic AESIs (≥ 25% in any arm) were hepatobiliary toxicity (premenopausal, 59.5% in the ribociclib arm versus 59.7% in the placebo arm; postmenopausal, 58.4% in both arms), QT interval prolongation (premenopausal, 49.4% vs. 6.5%; postmenopausal, 27.3% vs. 5.2%), and infections (premenopausal, 26.6% vs. 10.4%; postmenopausal, 35.1% vs. 31.2%). Hepatobiliary toxicity AESIs predominantly included increased AST and ALT (Table 3). Events requiring dose adjustment and/or interruption were reported in 9 patients per arm (11.4% with ribociclib vs. 11.7% with placebo) in the premenopausal cohort and 22 (28.6%) versus 9 (11.7%) in the postmenopausal cohort. In the premenopausal cohort, 0% of patients in the ribociclib arm versus 2.6% in the placebo arm discontinued study treatment due to hepatobiliary toxicity AESIs, which in the postmenopausal cohort occurred in 9.1% versus 3.9%.

Notable ECG changes from baseline are reported in Table S6. In the premenopausal cohort, a postbaseline Fredericia‐corrected QT interval (QTcF) > 480 ms occurred in 16.7% of patients in the ribociclib arm versus 2.7% in the placebo arm. Among these patients, 7.7% in the ribociclib arm versus 2.7% in the placebo arm experienced a postbaseline QTcF interval > 500 ms. An increase from baseline of > 60 ms in the QTcF interval was observed in 19.2% of patients in the ribociclib arm versus 2.7% in the placebo arm. In the postmenopausal cohort, postbaseline QTcF > 480 ms occurred in 10.5% of patients in the ribociclib arm versus 0% in the placebo arm. QTcF events of > 500 ms occurred in 2.6% of patients in the ribociclib arm versus 0% in the placebo arm. An increase from baseline > 60 ms in the QTcF interval occurred in 7.9% of patients in the ribociclib arm versus 0% in the placebo arm.

Data on notable hepatic values are shown in Table S7. The incidences of increased transaminases (> 3 × upper limit of normal [ULN], AST or ALT) in the ribociclib and placebo arms, respectively, were 11.5% versus 14.5% (premenopausal cohort) and 26.0% versus 13.0% (postmenopausal cohort). Total bilirubin increases > 2 × ULN were seen in 0 versus 1 patients in the ribociclib versus placebo arms (premenopausal cohort) and in 4 patients per arm (postmenopausal cohort). Concurrent elevations in transaminases (> 3 × ULN) and total bilirubin (> 2 × ULN) were 0% versus 1.3% (premenopausal cohort) and 3.9% versus 2.6% (postmenopausal cohort). No patients in the premenopausal cohort and 4 in the postmenopausal cohort (ribociclib arm, *n* = 3; placebo, *n* = 1) met the biochemical criteria for Hy's law.

The median time to first occurrence of and duration of grade ≥ 2 neutropenia, grade ≥ 3 ALT/AST elevation, and grade ≥ 2 QT prolongation are reported in Table S8.

## 4 DISCUSSION

This randomized phase 2 study of ribociclib + ET versus placebo + ET met its primary PFS objective in both pre‐ and postmenopausal cohorts of women with HR+/HER2− ABC. In the premenopausal cohort, the study resulted in an estimated 33% relative reduction in the risk of progression or death (HR 0.67 [95% CI: 0.45, 1.01]) in favor of the ribociclib arm and a 12.9‐month prolongation of median PFS. In the postmenopausal cohort, the study resulted in an estimated 60% relative reduction in the risk of progression or death (HR 0.40 [95% CI: 0.26, 0.62]) favoring the ribociclib arm, with the median PFS not yet reached. PFS results favoring the ribociclib versus placebo arms were largely consistent across subgroups. ORR was higher and median DOR longer in the ribociclib versus placebo arms in both cohorts. AEs in this Chinese population were manageable, and the overall safety profiles of ribociclib were comparable to those from the global studies, with a few specific safety events such as ECG QT prolongation and hepatobiliary toxicity (primarily increased AST/ALT) observed at higher frequencies.

Efficacy results from this study generally corroborated findings from the global pivotal studies, the most analogous of which were MONALEESA‐7 and ‐2, although cross‐trial comparisons should be considered with caution due to differences in study design and populations. PFS results from premenopausal patients showed benefit with ribociclib, as seen in MONALEESA‐7; PFS HR was 0.67 (95% CI: 0.45, 1.01) in this study (*N* = 156) versus 0.55 (95% CI: 0.44, 0.69) in the MONALEESA‐7 ITT population (*N* = 672) and 0.40 (95% CI: 0.26, 0.63) in its Asian subgroup (*n* = 198)^15^ (and 0.47 [95% CI: 0.31, 0.71] in an updated analysis [NSAI subpopulation, *n* = 166]).^17^ Likewise, PFS in postmenopausal patients was consistent with that in MONALEESA‐2, including the substantial improvement in the Asian subgroup; PFS HR was 0.40 (95% CI: 0.26, 0.62) in this study (*N* = 154) versus 0.56 (95% CI: 0.43, 0.72) in the MONALEESA‐2 ITT population (*N* = 668) and 0.39 (95% CI: 0.17, 0.91) in its Asian subgroup (*n* = 51).^16^

The median treatment durations with ribociclib were longer in this study compared with those from the corresponding MONALEESA studies at the time of primary analysis (e.g., in the ribociclib arms: premenopausal, 18.5 months in this study vs. 15.2 months in MONALEESA‐7; postmenopausal, 24.9 months in this study vs. 13.0 months in MONALEESA‐2).^15^, ^16^ While dose reductions were more frequent in the ribociclib arm versus placebo arm, they were consistent with data from the MONALEESA studies (41%–43% in this study had ≥ 1 dose reduction vs. 46% in a pooled safety analysis from MONALEESA‐2, −3, and ‐7^18^), and efficacy did not appear to be impacted. These observations indicate that effective management of dose‐dependent toxicity can be achieved without compromising the efficacy benefit of ribociclib.

The most common AEs in this study were hematologic (mostly neutropenia), similar to the global studies.^15^, ^16^ Grade 1/2 neutropenia occurred at somewhat higher frequencies than in MONALEESA‐7 and ‐2, although grade 3/4 frequencies were comparable.^15^, ^16^ Similarly, the MONARCH plus study of abemaciclib + NSAI in a cohort of predominantly postmenopausal Chinese patients showed increased AE frequency of mostly lower‐grade neutropenia relative to the global MONARCH 2 and 3 studies.^19^, ^20^, ^21^ PALOMA‐4, a study of palbociclib + letrozole in Asian postmenopausal patients, also showed higher neutropenia frequencies (all grade and grade 3/4) compared with the global PALOMA‐1‐3 studies.^22^, ^23^ In the current study, the median time to onset of grade ≥2 neutropenia in the ribociclib arm was approximately 2 weeks, with a similar median duration; in contrast, pooled analyses from the MONALEESA studies showed that despite a similar median time to grade ≥ 2 neutropenia, the median duration was approximately 4 weeks,^24^ suggesting that the neutropenia AE resolution may be more rapid in these Chinese patients relative to the pooled, global study populations.

Another notable observation was the incidence of prolonged ECG QT, especially in premenopausal patients. For instance, prolonged ECG QT occurred in 49% of ribociclib‐arm patients in this study's premenopausal cohort versus approximately 10% of ribociclib‐arm patients in MONALEESA‐7's ITT population. These AEs primarily presented as low‐grade events, with most not requiring treatment. In this study, the median time to grade ≥ 2 QT prolongation in the ribociclib arms was approximately 2 weeks, with a median duration of ≤ 1.6 weeks, while in the pooled MONALEESA analysis, median time to and duration of grade ≥ 2 QT prolongation were both approximately 2 weeks.^24^ No torsades de pointes, sudden death, or arrhythmia were reported, and all QT prolongation events were completely resolved after dose modification guidance per protocol.

Findings around hepatotoxicity should also be considered, with the predominant hepatobiliary AESIs, increased ALT or AST, seen in 35%–40% of ribociclib‐arm patients in this study versus 10%–15% in MONALEESA‐7 and ‐2.^15^, ^16^ One grade 5 AE occurred in the ribociclib + letrozole arm (hepatic failure in a patient with disease progression in the liver). In the ribociclib arm of the MONALEESA‐7 NSAI‐treated Asian subgroup, intermediate incidences of all‐grade increased ALT or AST (17% each) were also seen, relative to the ITT population.^17^ Similarly, increased all‐grade AST and ALT were observed in postmenopausal Asian patients (from Hong Kong and Singapore) with HR+/HER2− ABC from the phase 1b MONALEESAsia study with ribociclib + ET (increased AST, 26%; increased ALT, 35%, in patients treated with ribociclib 600 mg + letrozole, *n* = 23).^25^ Of note, increased ALT/AST incidences were similar between arms in this study but more skewed toward the treatment arms in the aforementioned ribociclib studies. Furthermore, in this study, the appearance of grade ≥ 3 AST/ALT elevations tended to be observed in the first 4–8 weeks overall, with variable median time to onset and median durations of 1–2 weeks. In pooled MONALEESA analyses, median onset of grade 3/4 AST/ALT elevations was 92 days in the ribociclib + ET arms, with a median duration of approximately 3 weeks.^26^ These data suggest quicker resolution in the Chinese population compared with global populations (1–2 vs. 3 weeks), similar to neutropenia.

The increased ALT/AST AE frequencies seen in this study's experimental arm were generally similar to those reported in MONARCH plus^19^ and PALOMA‐4,^23^ although the balance of all‐grade events between arms differed in each study (with generally similar rates between arms in PALOMA‐4, as seen in this study). Mirroring trends seen with neutropenia, these ALT/AST AEs had a higher frequency in MONARCH plus compared with MONARCH 2 and 3,^19^, ^20^, ^21^ while increased ALT/AST AE frequencies were not reported in the PALOMA‐1‐3 primary analyses, precluding comparisons.^27^, ^28^, ^29^ Collectively, these data suggest that evaluation of liver function data between Asian and global trials is complex, likely involving the influence of other confounding factors.

Evaluation of therapeutic agents in their target populations is important since ethnic and regional contributions can limit extrapolation of data from one population to another. Overall, this study's patient population represented the intended population from China, although higher disease burden and other associated risk factors were present compared with patients from MONALEESA‐7 and ‐2. For instance, 40%–45% had baseline ECOG PS 1, and approximately 50% had ≥3 metastatic sites, compared with approximately 25% and 35%, respectively, in MONALEESA‐7 and approximately 40% and 35% in MONALEESA‐2.^15^, ^16^ Despite these population differences, the overall study results are generally consistent with those of the Asian subgroup analyses from the global studies,^15^, ^16^ although specific toxicities, mainly low‐grade events, were seen at numerically higher rates than in prior ribociclib studies. This observation suggests the influence of intrinsic and/or extrinsic factors affecting AE rates in this population. Still, these data are encouraging given the unique epidemiology of patients with breast cancer in China (e.g., relatively young age,^2^ aggressive disease^30^) and the need for clinically meaningful treatment options in both the pre‐ and postmenopausal settings.

The study had several limitations. It was not powered to show statistical significance, although it was adequately sized to demonstrate the consistency of results in the Chinese populations compared with populations in prior global studies. The study was also conducted during the global COVID‐19 pandemic, and conduct was modified based on this. However, the modifications implemented were related to operational aspects, with minimal impact on the overall integrity or outcome of the study.

In conclusion, this China‐based phase 2 study of ribociclib + ET in women with HR+/HER2− metastatic breast cancer met its primary PFS objective in the pre‐ and postmenopausal cohorts. The overall safety profile of ribociclib was generally manageable, with no new safety signals observed in either cohort compared with the MONALEESA studies. Collectively, these results complement those reported in Asian patients from global pivotal studies, providing clinical evidence for the use of ribociclib + ET in Chinese patients with HR+/HER2− ABC, supporting approvals for this indication in China.

## AUTHOR CONTRIBUTIONS

**Zhimin Shao:** Conceptualization (equal); investigation (equal); supervision (equal); visualization (equal); writing – original draft (equal); writing – review and editing (equal). **Zhongsheng Tong:** Conceptualization (equal); investigation (equal); supervision (equal); visualization (equal); writing – review and editing (equal). **Qiang Liu:** Conceptualization (equal); investigation (equal); supervision (equal); visualization (equal); writing – review and editing (equal). **Wei Li:** Conceptualization (equal); investigation (equal); supervision (equal); visualization (equal); writing – review and editing (equal). **Li Cai:** Conceptualization (equal); investigation (equal); supervision (equal); visualization (equal); writing – review and editing (equal). **Kunwei Shen:** Conceptualization (equal); investigation (equal); supervision (equal); visualization (equal); writing – review and editing (equal). **Huiping Li:** Conceptualization (equal); investigation (equal); supervision (equal); visualization (equal); writing – review and editing (equal). **Chuan Wang:** Conceptualization (equal); investigation (equal); supervision (equal); visualization (equal); writing – review and editing (equal). **Jin Yang:** Conceptualization (equal); investigation (equal); supervision (equal); visualization (equal); writing – review and editing (equal). **Zhenchuan Song:** Conceptualization (equal); investigation (equal); methodology (equal); supervision (equal); visualization (equal); writing – review and editing (equal). **Shui Wang:** Conceptualization (equal); investigation (equal); methodology (equal); supervision (equal); visualization (equal); writing – review and editing (equal). **Ting Luo:** Conceptualization (equal); methodology (equal); supervision (equal); visualization (equal); writing – review and editing (equal). **Wenhe Zhao:** Conceptualization (equal); investigation (equal); methodology (equal); supervision (equal); visualization (equal); writing – review and editing (equal). **Haibo Wang:** Conceptualization (equal); investigation (equal); methodology (equal); supervision (equal); visualization (equal); writing – review and editing (equal). **Yueyin Pan:** Conceptualization (equal); investigation (equal); methodology (equal); supervision (equal); visualization (equal); writing – review and editing (equal). **Jianyun Nie:** Conceptualization (equal); investigation (equal); supervision (equal); visualization (equal); writing – review and editing (equal). **Xiaohua Zeng:** Conceptualization (equal); investigation (equal); supervision (equal); visualization (equal); writing – review and editing (equal). **Yanqing Bai:** Conceptualization (equal); data curation (equal); formal analysis (equal); methodology (equal); project administration (equal); resources (equal); supervision (equal); validation (equal); visualization (equal); writing – review and editing (equal). **Wendy Chiang:** Conceptualization (equal); data curation (equal); formal analysis (equal); methodology (equal); project administration (equal); resources (equal); supervision (equal); validation (equal); visualization (equal); writing – review and editing (equal). **Valeria Guarnaccia:** Conceptualization (equal); data curation (equal); formal analysis (equal); methodology (equal); project administration (equal); resources (equal); supervision (equal); validation (equal); visualization (equal); writing – review and editing (equal). **Yu Bi:** Conceptualization (equal); data curation (equal); formal analysis (equal); methodology (equal); project administration (equal); resources (equal); supervision (equal); validation (equal); visualization (equal); writing – review and editing (equal). **Binghe Xu:** Conceptualization (equal); investigation (equal); supervision (equal); visualization (equal); writing – original draft (equal).

## FUNDING INFORMATION

The study was supported by Novartis.

## CONFLICT OF INTEREST STATEMENT

Zhimin Shao, Zhongsheng Tong, Qiang Liu, Wei Li, Li Cai, Kunwei Shen, Huiping Li, Chuan Wang, Jin Yang, Zhenchuan Song, Shui Wang, Ting Luo, Wenhe Zhao, Haibo Wang, Yueyin Pan, Jianyun Nie, Xiaohua Zeng, and Binghe Xu have nothing to disclose. Yanqing Bai, Wendy Chiang, and Yu Bi report employment from Novartis. Valeria Guarnaccia reports employment and stock ownership from Novartis.

## ETHICS STATEMENT

The study was conducted according to ICH E6 Guideline for Good Clinical Practice and the Declaration of Helsinki. The study protocol and all amendments were approved by an independent ethics committee or institutional review board at each center. Patients were required to provide written informed consent. This study was registered at ClinicalTrials.gov (NCT03671330).

## Supporting information


Tables S1–S8.

## Data Availability

Novartis is committed to sharing with qualified external researchers access to patient‐level data and supporting clinical documents from eligible studies. These requests are reviewed and approved by an independent review panel on the basis of scientific merit. All data provided are anonymized to respect the privacy of patients who have participated in the trial in line with applicable laws and regulations.

## References

[1] Sung H , Ferlay J , Siegel RL , et al. Global cancer statistics 2020: GLOBOCAN estimates of incidence and mortality worldwide for 36 cancers in 185 countries. CA Cancer J Clin. 2021;71:209‐249. 33538338 10.3322/caac.21660

[2] Tao X , Li T , Gandomkar Z , Brennan PC , Reed WM . Incidence, mortality, survival, and disease burden of breast cancer in China compared to other developed countries. Asia Pac J Clin Oncol. 2023;19:645‐654. 37026375 10.1111/ajco.13958

[3] Xia C , Dong X , Li H , et al. Cancer statistics in China and United States, 2022: profiles, trends, and determinants. Chin Med J. 2022;135:584‐590. 35143424 10.1097/CM9.0000000000002108 PMC8920425

[4] Luo C , Li N , Lu B , et al. Global and regional trends in incidence and mortality of female breast cancer and associated factors at national level in 2000 to 2019. Chin Med J. 2022;135:42‐51. 34593698 10.1097/CM9.0000000000001814 PMC8850868

[5] Chen Z , Ouyang Q , Wang Y , et al. Real‐world first‐line treatment patterns and outcomes in hormone receptor‐positive advanced breast cancer patients: a multicenter, retrospective study in China. Front Oncol. 2022;12:829693. 35311126 10.3389/fonc.2022.829693 PMC8928103

[6] Howlader N , Altekruse SF , Li CI , et al. US incidence of breast cancer subtypes defined by joint hormone receptor and HER2 status. J Natl Cancer Inst. 2014;106:dju055. 24777111 10.1093/jnci/dju055 PMC4580552

[7] Murali B , Durbin L , Vijaykumar S , et al. Treatment of HR+/HER2‐ breast cancer in urban mainland China: results from the CancerMPact survey 2019. Breast Cancer Res Treat. 2022;195:441‐451. 35986800 10.1007/s10549-022-06709-x PMC9464725

[8] Zuo T , Zeng H , Li H , et al. The influence of stage at diagnosis and molecular subtype on breast cancer patient survival: a hospital‐based multi‐center study. Chin J Cancer. 2017;36:84. 29070080 10.1186/s40880-017-0250-3 PMC5657106

[9] Jiang Z , Li J , Chen J , et al. Chinese Society of Clinical Oncology (CSCO) breast cancer guidelines 2022. Transl Breast Cancer Res. 2022;3:13. 38751537 10.21037/tbcr-22-21 PMC11093004

[10] Hanker AB , Sudhan DR , Arteaga CL . Overcoming endocrine resistance in breast cancer. Cancer Cell. 2020;37:496‐513. 32289273 10.1016/j.ccell.2020.03.009 PMC7169993

[11] National Medical Products Administration . Imported Drugs—Basic Information of “Guoyao Zhunzi HJ20230003.”. 2023. https://www.nmpa.gov.cn/datasearch/search‐info.html?nmpa=aWQ9Yjc2YTg2YzI2ZmYxYWNlYmNiMDQxZDdjMDI1MWQ0YTUmaXRlbUlkPWZmODA4MDgxODNjYWQ3NTAwMTg0MDg4NjY1NzExODAw

[12] Slamon DJ , Neven P , Chia S , et al. Overall survival with ribociclib plus fulvestrant in advanced breast cancer. N Engl J Med. 2020;382:514‐524. 31826360 10.1056/NEJMoa1911149

[13] Im SA , Lu YS , Bardia A , et al. Overall survival with ribociclib plus endocrine therapy in breast cancer. N Engl J Med. 2019;381:307‐316. 31166679 10.1056/NEJMoa1903765

[14] Hortobagyi GN , Stemmer SM , Burris HA , et al. Overall survival with ribociclib plus letrozole in advanced breast cancer. N Engl J Med. 2022;386:942‐950. 35263519 10.1056/NEJMoa2114663

[15] Tripathy D , Im SA , Colleoni M , et al. Ribociclib plus endocrine therapy for premenopausal women with hormone‐receptor‐positive, advanced breast cancer (MONALEESA‐7): a randomised phase 3 trial. Lancet Oncol. 2018;19:904‐915. 29804902 10.1016/S1470-2045(18)30292-4

[16] Hortobagyi GN , Stemmer SM , Burris HA , et al. Ribociclib as first‐line therapy for HR‐positive, advanced breast cancer. N Engl J Med. 2016;375:1738‐1748. 27717303 10.1056/NEJMoa1609709

[17] Lu YS , Sohn J , Lee KS , et al. Efficacy and quality of life in premenopausal Asian patients with hormone receptor–positive, HER2‐negative advanced breast cancer treated in the MONALEESA‐7 study. Ann Oncol. 2020;31:S1260.

[18] Burris HA , Chan A , Bardia A , et al. Safety and impact of dose reductions on efficacy in the randomised MONALEESA‐2, −3 and −7 trials in hormone receptor‐positive, HER2‐negative advanced breast cancer. Br J Cancer. 2021;125:679‐686. 34158598 10.1038/s41416-021-01415-9 PMC8405616

[19] Zhang QY , Sun T , Yin YM , et al. MONARCH plus: abemaciclib plus endocrine therapy in women with HR+/HER2‐ advanced breast cancer: the multinational randomized phase III study. Ther Adv Med Oncol. 2020;12:1758835920963925. 33149768 10.1177/1758835920963925 PMC7586037

[20] Goetz MP , Toi M , Campone M , et al. MONARCH 3: abemaciclib as initial therapy for advanced breast cancer. J Clin Oncol. 2017;35:3638‐3646. 28968163 10.1200/JCO.2017.75.6155

[21] Sledge GW Jr , Toi M , Neven P , et al. MONARCH 2: abemaciclib in combination with fulvestrant in women with HR+/HER2‐ advanced breast cancer who had progressed while receiving endocrine therapy. J Clin Oncol. 2017;35:2875‐2884. 28580882 10.1200/JCO.2017.73.7585

[22] Dieras V , Rugo HS , Schnell P , et al. Long‐term pooled safety analysis of palbociclib in combination with endocrine therapy for HR+/HER2‐ advanced breast cancer. J Natl Cancer Inst. 2019;111:419‐430. 30032196 10.1093/jnci/djy109 PMC6449170

[23] Xu B , Hu X , Li W , et al. Palbociclib plus letrozole versus placebo plus letrozole in Asian postmenopausal women with oestrogen receptor‐positive/human epidermal growth factor receptor 2‐negative advanced breast cancer: primary results from PALOMA‐4. Eur J Cancer. 2022;175:236‐245. 36155117 10.1016/j.ejca.2022.08.012

[24] Burris HA , Chan A , Im S‐A , et al. Abstract P6‐18‐15: Ribociclib+ endocrine therapy in hormone receptor‐positive, HER2‐negative advanced breast cancer: a pooled safety analysis. Presented at the 2018 San Antonio Breast Cancer Symposium. December 4‐8, 2018. Poster presenation (publication number P6‐18‐15).

[25] Yap YS , Chiu J , Ito Y , et al. Ribociclib, a CDK 4/6 inhibitor, plus endocrine therapy in Asian women with advanced breast cancer. Cancer Sci. 2020;111:3313‐3326. 32619077 10.1111/cas.14554 PMC7469771

[26] KISKALI (ribociclib) . Summary of product characteristics. 2022. https://www.ema.europa.eu/en/documents/product‐information/kisqali‐epar‐product‐information_en.pdf

[27] Finn RS , Martin M , Rugo HS , et al. Palbociclib and letrozole in advanced breast cancer. N Engl J Med. 2016;375:1925‐1936. 27959613 10.1056/NEJMoa1607303

[28] Finn RS , Crown JP , Lang I , et al. The cyclin‐dependent kinase 4/6 inhibitor palbociclib in combination with letrozole versus letrozole alone as first‐line treatment of oestrogen receptor‐positive, HER2‐negative, advanced breast cancer (PALOMA‐1/TRIO‐18): a randomised phase 2 study. Lancet Oncol. 2015;16:25‐35. 25524798 10.1016/S1470-2045(14)71159-3

[29] Turner NC , Ro J , André F , et al. Palbociclib in hormone‐receptor‐positive advanced breast cancer. N Engl J Med. 2015;373:209‐219. 26030518 10.1056/NEJMoa1505270

[30] Azim HA Jr , Partridge AH . Biology of breast cancer in young women. Breast Cancer Res. 2014;16:427. 25436920 10.1186/s13058-014-0427-5 PMC4303229

