# Correction to: Initial ribociclib plus endocrine therapy for HR+/HER2− advanced breast cancer in pre‐ and postmenopausal Chinese women: Primary results from a phase 2 randomized study

**DOI:** 10.1002/cam4.70233

**Published:** 2024-09-14

Shao Z, et al., *Cancer Med*. 2024 Aug;13(15):e7408. https://doi.org/10.1002/cam4.7408. PMID: 39136200; PMCID: PMC11320080

We requested post‐publication to add the following line after the first line in the acknowledgment section:

We also thank DMC chair Prof. Xichun Hu, DMC member Prof. Jin Zhang, Prof. Shusen Wang, Prof. Renbing Liu, and Prof. Chen Yao for their contributions to the trial.

We apologize for this error.

