# Supplementary material for: Initial ribociclib plus endocrine therapy for HR+/HER2− advanced breast cancer in pre‐ and postmenopausal Chinese women: Primary results from a phase 2 randomized study
Source: Cancer Med. 2024 Aug 13;13(15):e7408. doi: 10.1002/cam4.7408 (PMC11320080; doi:10.1002/cam4.7408)
Supplement: Supplementary file 1 — Tables S1–S8. [file CAM4-13-e7408-s001.docx]

**Supplementary Material**

**Table S1. Investigators and study sites**

| Principal investigator | Site name | Site location |
| --- | --- | --- |
| Zhimin Shao | Fudan University Shanghai Cancer Center | Shanghai |
| Binghe Xu | Cancer Institute and Hospital, Chinese Academy of Medical Science | Beijing |
| Qiang Liu | Sun Yat-Sen Memorial Hospital, Sun Yat-Sen University | Guangzhou |
| Zhongsheng Tong | Tianjin Medical University Cancer institute and Hospital | Tianjin |
| Li Cai | Harbin Medical University Cancer Hospital | Harbin |
| Jian Huang | 2nd Affiliated Hospital of Zhejiang University School of Medicine | Hangzhou |
| Wei Li | 1st Affiliated Hospital of Jilin University | Chang Chun |
| Huiping Li | Peking University Beijing Cancer Hospital & Institute | Beijing |
| Ting Luo | West China Hospital, Sichuan University | Chengdu |
| Jianyun Nie | Yunnan Provincial Cancer Hospital | Kunming |
| Quchang Ouyang | Cancer Hospital of Hunan Province | Changsha |
| Yueyin Pan | Anhui Provincial Hospital | Hefei |
| Kunwei Shen | Ruijin Hospital Shanghai Jiao Tong University School of Medicine | Shanghai |
| Zhenchuan Song | 4th Hospital of Hebei Medical University | Shijiazhuang |
| Tao Sun | Liaoning Cancer Hospital | Shengyang |
| Chuan Wang | Affiliated Union Hospital of Fujian Medical University | Fuzhou |
| Haibo Wang | The Affiliated Hospital of Qingdao University | Qingdao |
| Hong Wang | The 3rd Hospital of Nanchang | Nanchang |
| Shu Wang | Peking University People’s Hospital | Beijing |
| Shui Wang | Jiang Su Province Renmin Hospital | Nanjing |
| Jin Yang | The 1st Affiliated Hospital of Xi'an Jiaotong University | Xian |
| Xiaohua Zeng | Chongqing Cancer Hospital | Chongqing |
| Wenhe Zhao | Sir Run Run Shaw Hospital/Zhejing University School of Medicine | Hangzhou |
| Zhendong Zheng | The General Hospital of Shenyang Military | Shengyang |
| Zhixiang Zhuang | The 2nd Affiliated Hospital of Suzhou University | Suzhou |

Includes all study steering committee members and investigators whose sites enrolled ≥1 patients.

**Table S2. Exposure and relative dose intensity^a^**

|  | **Study treatment** | | **Ribociclib/placebo** | | **Anastrozole** | | **Letrozole** | | **Goserelin** | |
| --- | --- | --- | --- | --- | --- | --- | --- | --- | --- | --- |
| **Premenopausal cohort** | **RIB + NSAI + G (n=79)** | **PBO + NSAI + G (n=77)** | **RIB + NSAI + G (n=79)** | **PBO + NSAI + G (n=77)** | **RIB + NSAI + G (n=16)** | **PBO + NSAI + G (n=14)** | **RIB + NSAI + G (n=63)** | **PBO + NSAI + G (n=63)** | **RIB + NSAI + G (n=79)** | **PBO + NSAI + G (n=77)** |
| Patients with indicated treatment duration, n (%) | | | |  |  |  |  |  |  |  |
| <3 mo | 19 (24.1) | 15 (19.5) | 20 (25.3) | 17 (22.1) | 6 (37.5) | 6 (42.9) | 13 (20.6) | 9 (14.3) | 19 (24.1) | 15 (19.5) |
| 3 to <6 mo | 3 (3.8) | 6 (7.8) | 5 (6.3) | 5 (6.5) | 1 (6.3) | 0 | 4 (6.3) | 6 (9.5) | 3 (3.8) | 6 (7.8) |
| 6 to <9 mo | 4 (5.1) | 4 (5.2) | 5 (6.3) | 5 (6.5) | 0 | 1 (7.1) | 2 (3.2) | 3 (4.8) | 4 (5.1) | 4 (5.2) |
| 9 to <12 mo | 6 (7.6) | 12 (15.6) | 3 (3.8) | 12 (15.6) | 1 (6.3) | 1 (7.1) | 6 (9.5) | 11 (17.5) | 6 (7.6) | 12 (15.6) |
| 12 to <15 mo | 2 (2.5) | 8 (10.4) | 2 (2.5) | 10 (13.0) | 0 | 2 (14.3) | 2 (3.2) | 8 (12.7) | 2 (2.5) | 8 (10.4) |
| 15 to <18 mo | 5 (6.3) | 4 (5.2) | 4 (5.1) | 3 (3.9) | 0 | 0 | 4 (6.3) | 2 (3.2) | 5 (6.3) | 4 (5.2) |
| ≥18 mo | 40 (50.6) | 28 (36.4) | 40 (50.6) | 25 (32.5) | 8 (50.0) | 4 (28.6) | 32 (50.8) | 24 (38.1) | 40 (50.6) | 28 (36.4) |
| Treatment duration, median (range), mo | 18.5  (0.9-42.9) | 13.8  (0.4-39.6) | 18.5  (0.3-42.9) | 11.3  (0-39.3) | 13.9  (1.7-42.9) | 8.3  (1.8-37.9) | 19.4  (0.3-41.5) | 13.0  (0 -39.6) | 18.5  (0.9-42.6) | 13.8  (0.4-39.6) |
| Relative dose intensity, median (range), % | 95.5  (39-100) | 100 (44-119) | — | — | — | — | — | — | — | — |
| **Postmenopausal cohort** | **RIB + LET (n=77)** | **PBO + LET (n=77)** | **RIB + LET (n=77)** | **PBO + LET (n=77)** | **RIB + LET (n=0)** | **PBO + LET (n=1)** | **RIB + LET (n=77)** | **PBO + LET (n=77)** | — | — |
| Patients with indicated treatment duration, n (%) | | | |  |  |  |  |  |  |  |
| <3 mo | 10 (13.0) | 12 (15.6) | 14 (18.2) | 12 (15.6) | — | 1 (100) | 10 (13.0) | 11 (14.5) | — | — |
| 3 to <6 mo | 3 (3.9) | 6 (7.8) | 1 (1.3) | 6 (7.8) | — | 0 | 3 (3.9) | 6 (7.9) | — | — |
| 6 to <9 mo | 4 (5.2) | 4 (5.2) | 4 (5.2) | 8 (10.4) | — | 0 | 4 (5.2) | 4 (5.3) | — | — |
| 9 to <12 mo | 4 (5.2) | 8 (10.4) | 4 (5.2) | 5 (6.5) | — | 0 | 4 (5.2) | 8 (10.5) | — | — |
| 12 to <15 mo | 2 (2.6) | 7 (9.1) | 2 (2.6) | 6 (7.8) | — | 0 | 2 (2.6) | 7 (9.2) | — | — |
| 15 to <18 mo | 4 (5.2) | 5 (6.5) | 4 (5.2) | 5 (6.5) | — | 0 | 4 (5.2) | 5 (6.5) | — | — |
| ≥18 mo | 50 (64.9) | 35 (45.5) | 48 (62.3) | 35 (45.5) | — | 0 | 50 (64.9) | 35 (45.5) | — | — |
| Treatment duration, median (range), mo | 24.9  (0.8-42.3) | 15.6  (0.6-39.1) | 24.9  (0.1-42.3) | 15.4  (0.6-39.1) | — | 1.8  (1.8-1.8) | 24.9  (0.8-42.3) | 16.1  (0.6-39.1) | — | — |
| Relative dose intensity, median (range), % | 95.0  (45-100) | 100  (74-105) | — | — | — | — | — | — | — | — |

G, goserelin; LET, letrozole; NSAI, nonsteroidal aromatase inhibitor; PBO, placebo; RIB, ribociclib.

^a^ Based on the safety-evaluable population.

# Table S3. Dose reductions, interruptions, and delays of ribociclib/placebo^a^

|  | **Premenopausal** |  | **Postmenopausal** |  |
| --- | --- | --- | --- | --- |
|  | **RIB + NSAI + G (n=79)** | **PBO + NSAI + G (n=77)** | **RIB + LET**  **(n=77)** | **PBO + LET (n=77)** |
| **Dose reductions, n (%)** |  |  |  |  |
| 0 | 47 (59.5) | 73 (94.8) | 44 (57.1) | 71 (92.2) |
| 1 | 26 (32.9) | 3 (3.9) | 23 (29.9) | 6 (7.8) |
| 2 | 6 (7.6) | 1 (1.3) | 10 (13.0) | 0 |
| **Patients with ≥1 dose reduction due to indicated reason, n (%)^b^** | **32 (40.5)** | **4 (5.2)** | **33 (42.9)** | **6 (7.8)** |
| AE | 31 (39.2) | 2 (2.6) | 32 (41.6) | 5 (6.5) |
| Missing | 3 (3.8) | 1 (1.3) | 1 (1.3) | 1 (1.3) |
| Physician decision | 0 | 1 (1.3) | 3 (3.9) | 0 |
| Dosing error | 0 | 0 | 1 (1.3) | 0 |
| **Dose interruptions, n (%)^b,c^** |  |  |  |  |
| 0 | 14 (17.7) | 44 (57.1) | 13 (16.9) | 47 (61.0) |
| 1 | 18 (22.8) | 17 (22.1) | 14 (18.2) | 12 (15.6) |
| 2 | 12 (15.2) | 11 (14.3) | 4 (5.2) | 9 (11.7) |
| ≥3 | 35 (44.3) | 5 (6.5) | 46 (59.7) | 9 (11.7) |
| **Patients with ≥1 dose interruption due to indicated reason, n (%)** | **65 (82.3)** | **33 (42.9)** | **64 (83.1)** | **30 (39.0)** |
| AE | 59 (74.7) | 15 (19.5) | 58 (75.3) | 14 (18.2) |
| Dosing error | 19 (24.1) | 20 (26.0) | 24 (31.2) | 18 (23.4) |
| Technical problems | 7 (8.9) | 5 (6.5) | 10 (13.0) | 4 (5.2) |
| Patient decision | 2 (2.5) | 2 (2.6) | 2 (2.6) | 1 (1.3) |
| Physician decision | 0 | 0 | 1 (1.3) | 2 (2.6) |
| Dispensing error | 0 | 0 | 0 | 1 (1.3) |
| **Dose delays, n (%)^d^** |  |  |  |  |
| 0 | 50 (63.3) | 69 (89.6) | 38 (49.4) | 63 (81.8) |
| 1 | 15 (19.0) | 4 (5.2) | 21 (27.3) | 8 (10.4) |
| 2 | 6 (7.6) | 3 (3.9) | 7 (9.1) | 2 (2.6) |
| ≥3 | 8 (10.1) | 1 (1.3) | 11 (14.3) | 4 (5.2) |
| **Patients with ≥1 dose delay due to indicated reason, n (%)** | 29 (36.7) | 8 (10.4) | 39 (50.6) | 14 (18.2) |
| AE | 25 (31.6) | 2 (2.6) | 28 (36.4) | 6 (7.8) |
| Dosing error | 10 (12.7) | 7 (9.1) | 16 (20.8) | 9 (11.7) |
| Technical problems | 1 (1.3) | 1 (1.3) | 3 (3.9) | 0 |
| Patient decision | 1 (1.3) | 0 | 0 | 0 |
| Physician decision | 0 | 0 | 1 (1.3) | 1 (1.3) |
| Dispensing error | 0 | 0 | 0 | 1 (1.3) |

AE, adverse event; G, goserelin; LET, letrozole; NSAI, nonsteroidal aromatase inhibitor; PBO, placebo; RIB, ribociclib.

^a^ Based on the safety-evaluable population.

^b^ Patients with multiple occurrences of a dose reduction or interruption for an indicated reason were only counted once in that category.
^c^ Interruptions were defined as a 0-mg dose entered in the case report form for ≥1 days with the dose interruption box checked. The dosing breaks for ribociclib/placebo on days 22-28 of each cycle were not counted as interruptions.
^d^ Delays were defined as interruptions that occurred at the beginning of a new cycle after the day 22-28 dosing break.

# Table S4. Safety summary^a^

|  | **Premenopausal cohort** | | | | **Postmenopausal cohort** | | | |
| --- | --- | --- | --- | --- | --- | --- | --- | --- |
| **Preferred term** | **RIB + NSAI + G (n=79)** | | **PBO + NSAI + G (n=77)** | | **RIB + LET (n=77)** | | **PBO + LET (n=77)** | |
| **Patients with AE, n (%)** | **All grades** | **Grade 3/4** | **All grades** | **Grade 3/4** | **All grades** | **Grade 3/4** | **All grades** | **Grade 3/4** |
| AE^b^ | 79 (100) | 61 (77.2) | 74 (96.1) | 21 (27.3) | 77 (100) | 62 (80.5) | 74 (96.1) | 32 (41.6) |
| TRAE^c^ | 79 (100) | 57 (72.2) | 69 (89.6) | 15 (19.5) | 77 (100) | 59 (76.6) | 66 (85.7) | 17 (22.1) |
| Serious AE | 12 (15.2) | 9 (11.4) | 3 (3.9) | 1 (1.3) | 16 (20.8) | 11 (14.3) | 14 (18.2) | 12 (15.6) |
| Serious TRAE | 7 (8.9) | 5 (6.3) | 2 (2.6) | 1 (1.3) | 8 (10.4) | 6 (7.8) | 4 (5.2) | 4 (5.2) |
| AE leading to discontinuation | 5 (6.3) | 3 (3.8) | 5 (6.5) | 2 (2.6) | 11 (14.3) | 6 (7.8) | 5 (6.5) | 3 (3.9) |
| TRAE leading to discontinuation of any treatment component | 4 (5.1) | 3 (3.8) | 4 (5.2) | 2 (2.6) | 11 (14.3) | 6 (7.8) | 4 (5.2) | 1 (1.3) |
| AE requiring dose interruption | 54 (68.4) | 46 (58.2) | 19 (24.7) | 5 (6.5) | 61 (79.2) | 52 (67.5) | 16 (20.8) | 11 (14.3) |
| TRAE requiring dose interruption | 53 (67.1) | 45 (57.0) | 16 (20.8) | 4 (5.2) | 60 (77.9) | 50 (64.9) | 13 (16.9) | 8 (10.4) |
| AE requiring dose adjustment | 30 (38.0) | 23 (29.1) | 3 (3.9) | 2 (2.6) | 31 (40.3) | 19 (24.7) | 5 (6.5) | 3 (3.9) |
| TRAE requiring dose adjustment | 30 (38.0) | 23 (29.1) | 3 (3.9) | 2 (2.6) | 31 (40.3) | 19 (24.7) | 5 (6.5) | 3 (3.9) |
| AE requiring additional therapy^d^ | 59 (74.7) | 43 (54.4) | 49 (63.6) | 11 (14.3) | 70 (90.9) | 47 (61.0) | 47 (61.0) | 22 (28.6) |
| TRAE requiring additional therapy | 54 (68.4) | 40 (50.6) | 34 (44.2) | 7 (9.1) | 64 (83.1) | 40 (51.9) | 38 (49.4) | 12 (15.6) |

AE, adverse event; G, goserelin; LET, letrozole; NSAI, nonsteroidal aromatase inhibitor; PBO, placebo; RIB, ribociclib; TRAE, treatment-related AE.

^a^ Includes AEs occurring up to 30 days after the last date of study treatment.

^b^ In the premenopausal cohort, 18 deaths (in 22.8% of patients) in the RIB arm and 23 (29.9%) in the PBO arm occurred, none of which occurred on treatment. In the postmenopausal cohort, 12 deaths (15.6% of patients) in the RIB arm and 20 (26.0%) in the PBO arm occurred, of which 1 per arm (1.3%) occurred on treatment. All deaths, but not on-treatment deaths, include those occurring >30 days after last date of study treatment.

^c^ TRAEs were defined as AEs suspected to be related to any component of study treatment.

^d^ Includes all nondrug therapy and concomitant medications.

# Table S5. AESIs irrespective of causality

| **AESI grouping, n (%)** | **Premenopausal cohort** | | | | | | **Postmenopausal cohort** | | | | | |
| --- | --- | --- | --- | --- | --- | --- | --- | --- | --- | --- | --- | --- |
|  | **RIB + NSAI + G (n=79)** | | | **PBO + NSAI + G (n=77)** | | | **RIB + LET (n=77)** | | | **PBO + LET (n=77)** | | |
|  | **All grades** | **Grade 3** | **Grade 4** | **All grades** | **Grade 3** | **Grade 4** | **All grades** | **Grade 3** | **Grade 4** | **All grades** | **Grade 3** | **Grade 4** |
| **Hematologic AESIs: Myelosuppression** |  |  |  |  |  |  |  |  |  |  |  |  |
| Leukopenia^a^ | 78 (98.7) | 26 (32.9) | 2 (2.5) | 36 (46.8) | 1 (1.3) | 0 | 73 (94.8) | 22 (28.6) | 1 (1.3) | 25 (32.5) | 2 (2.6) | 0 |
| Neutropenia^b^ | 78 (98.7) | 37 (46.8) | 12 (15.2) | 23 (29.9) | 3 (3.9) | 0 | 74 (96.1) | 44 (57.1) | 5 (6.5) | 16 (20.8) | 1 (1.3) | 0 |
| Anemia^c^ | 52 (65.8) | 5 (6.3) | 0 | 13 (16.9) | 1 (1.3) | 0 | 42 (54.5) | 3 (3.9) | 0 | 11 (14.3) | 5 (6.5) | 0 |
| Thrombocytopenia^d^ | 31 (39.2) | 1 (1.3) | 1 (1.3) | 12 (15.6) | 1 (1.3) | 0 | 30 (39.0) | 1 (1.3) | 0 | 14 (18.2) | 0 | 0 |
| Other^e^ | 0 | 0 | 0 | 1 (1.3) | 1 (1.3) | 0 | 0 | 0 | 0 | 0 | 0 | 0 |
| **Nonhematologic AESIs^f^** |  |  |  |  |  |  |  |  |  |  |  |  |
| Hepatobiliary toxicity^g^ | 47 (59.5) | 10 (12.7) | 0 | 46 (59.7) | 5 (6.5) | 1 (1.3) | 45 (58.4) | 14 (18.2) | 0 | 45 (58.4) | 9 (11.7) | 2 (2.6) |
| QT interval prolongation^h^ | 39 (49.4) | 4 (5.1) | 0 | 5 (6.5) | 2 (2.6) | 0 | 21 (27.3) | 1 (1.3) | 1 (1.3) | 4 (5.2) | 0 | 0 |
| Infections^i^ | 21 (26.6) | 3 (3.8) | 0 | 8 (10.4) | 0 | 0 | 27 (35.1) | 3 (3.9) | 0 | 24 (31.2) | 1 (1.3) | 1 (1.3) |
| Renal toxicity^j^ | 16 (20.3) | 1 (1.3) | 0 | 5 (6.5) | 0 | 0 | 15 (19.5) | 0 | 0 | 6 (7.8) | 0 | 0 |
| Second primary  malignancies^k^ | 0 | 0 | 0 | 0 | 0 | 0 | 1 (1.3) | 0 | 1 (1.3) | 1 (1.3) | 1 (1.3) | 0 |

AESI, adverse event of special interest; G, goserelin; LET, letrozole; NSAI, nonsteroidal aromatase inhibitor; PBO, placebo; RIB, ribociclib.

^a^ Includes decreased white blood cell count, leukopenia, decreased lymphocyte count, or lymphopenia (observed preferred terms).

^b^ Includes neutropenia, febrile neutropenia, or decreased neutrophil count (observed preferred terms).

^c^ Includes anemia, decreased hemoglobin, erythropenia, or decreased red blood cell count (observed preferred terms).

^d^ Includes thrombocytopenia or decreased platelet count (observed preferred terms).

^e^ Includes myelosuppression (observed preferred term).

^f^ Additionally, 1 grade 5 hepatobiliary toxicity event (hepatic failure) occurred in 1 patient in the ribociclib arm of the postmenopausal cohort.

^g^ Includes increased alanine aminotransferase, increased aspartate aminotransferase, increased gamma-glutamyltransferase, increased blood alkaline phosphatase, increased blood bilirubin, abnormal hepatic function, hypoalbuminemia, increased conjugated bilirubin, increased unconjugated blood bilirubin, hepatic failure, drug-induced liver injury, increased ammonia, or decreased blood fibrinogen (observed preferred terms).

^h^ Includes QT prolonged, Ventricular arrhythmia, or syncope (observed preferred terms).

^I^ Includes upper respiratory tract infection, urinary tract infection, nasopharyngitis, pneumonia, conjunctivitis, cystitis, bronchitis, influenza, pharyngitis, eye infection, gastroenteritis, gingivitis, lymphangitis, herpes virus infection, herpes zoster, oral herpes, rhinitis, urethritis, appendicitis perforated, biliary tract infection, bronchitis, chronic gastritis, pulpitis dental, or wound infection (observed preferred terms).

^j^ Includes increased blood creatinine, increased blood urea, decreased glomerular filtration rate, renal impairment, or acute kidney injury (observed preferred terms).

^k^ Includes acute promyelocytic leukemia or papillary thyroid cancer (observed preferred terms).

# Table S6. Notable ECG changes from baseline

|  | **Premenopausal** | | **Postmenopausal** | |
| --- | --- | --- | --- | --- |
| **n/n (%)** | **RIB + NSAI + G (n=79)** | **PBO + NSAI + G (n=77)** | **RIB + LET  (n=77)** | **PBO + LET  (n=77)** |
| QTcF^a^ |  |  |  |  |
| New >450 ms | 44/78 (56.4) | 6/75 (8.0) | 32/76 (42.1) | 10/77 (13.0) |
| New >480 ms | 13/78 (16.7) | 2/75 (2.7) | 8/76 (10.5) | 0/77 |
| New >500 ms | 6/78 (7.7) | 2/75 (2.7) | 2/76 (2.6) | 0/77 |
| Increase from baseline QTcF^b^ |  |  |  |  |
| >30 ms | 47/78 (60.3) | 5/75 (6.7) | 41/76 (53.9) | 5/77 (6.5) |
| >60 ms | 15/78 (19.2) | 2/75 (2.7) | 6/76 (7.9) | 0/77 |

All scheduled and unscheduled visits were included in this analysis. Baseline was defined as the average of last ECG measurements taken before start of study treatment.

ECG, electrocardiogram; G, goserelin; LET, letrozole; NSAI, nonsteroidal aromatase inhibitor; PBO, placebo; QTcF, Fredericia-corrected QT interval; RIB, ribociclib.

^a^ Percentages were calculated based on the number of patients with both baseline and postbaseline evaluations as well as those with baseline evaluations not meeting the criteria.

^b^ Percentages were calculated based on the number of patients with both baseline and postbaseline evaluations.

# Table S7. Patients with notable hepatic values

| **n/N (%)** | **Premenopausal cohort** | | | **Postmenopausal cohort** | | | |
| --- | --- | --- | --- | --- | --- | --- | --- |
|  | **RIB + NSAI + G (n=79)** | **PBO + NSAI + G (n=77)** | | **RIB + LET**  **(n=77)** | | **PBO + LET**  **(n=77)** | |
| AST |  |  | |  | |  | |
| >3 × ULN | 6/78 (7.7) | 5/76 (6.6) | | 13/77 (16.9) | | 7/77 (9.1) | |
| >5 × ULN | 2/78 (2.6) | 2/76 (2.6) | | 9/77 (11.7) | | 3/77 (3.9) | |
| >8 × ULN | 0/78 | 1/76 (1.3) | | 5/77 (6.5) | | 1/77 (1.3) | |
| >10 × ULN | 0/78 | 1/76 (1.3) | | 5/77 (6.5) | | 1/77 (1.3) | |
| >20 × ULN | 0/78 | 0/76 | | 1/77 (1.3) | | 0/77 | |
| ALT |  |  | |  | |  | |
| >3 × ULN | 7/78 (9.0) | 9/76 (11.8) | | 20/77 (26.0) | | 5/77 (6.5) | |
| >5 × ULN | 2/78 (2.6) | 2/76 (2.6) | | 12/77 (15.6) | | 4/77 (5.2) | |
| >8 × ULN | 0/78 | 0/76 | | 5/77 (6.5) | | 0/77 | |
| >10 × ULN | 0/78 | 0/76 | | 3/77 (3.9) | | 0/77 | |
| AST or ALT |  |  | |  | |  | |
| >3 × ULN | 9/78 (11.5) | 11/76 (14.5) | | 20/77 (26.0) | | 10/77 (13.0) | |
| >5 × ULN | 3/78 (3.8) | 3/76 (3.9) | | 13/77 (16.9) | | 5/77 (6.5) | |
| >8 × ULN | 0/78 | 1/76 (1.3) | | 7/77 (9.1) | | 1/77 (1.3) | |
| >10 × ULN | 0/78 | 1/76 (1.3) | | 5/77 (6.5) | | 1/77 (1.3) | |
| >20 × ULN | 0/78 | 0/76 | | 1/77 (1.3) | | 0/77 | |
| TBL |  |  | |  | |  | |
| >ULN | 9/78 (11.5) | 8/76 (10.5) | | 14/77 (18.2) | | 13/77 (16.9) | |
| >2 × ULN | 0/78 | 1/76 (1.3) | | 4/77 (5.2) | | 4/77 (5.2) | |
| ALP |  |  | |  | |  | |
| >1.5 × ULN | 12/79 (15.2) | 13/76 (17.1) | | 13/77 (16.9) | | 15/77 (19.5) | |
| >2 × ULN | 8/79 (10.1) | 8/76 (10.5) | | 7/77 (9.1) | | 11/77 (14.3) | |
| >3 × ULN | 4/79 (5.1) | 3/76 (3.9) | | 3/77 (3.9) | | 6/77 (7.8) | |
| >5 × ULN | 1/79 (1.3) | 2/76 (2.6) | | 0/77 | | 2/77 (2.6) | |
| >8 × ULN | 1/79 (1.3) | 0/76 | | 0/77 | | 2/77 (2.6) | |
| >10 × ULN | 1/79 (1.3) | 0/76 | | 0/77 | | 0/77 | |
| ALT or AST and total bilirubin |  |  | |  | |  | |
| AT >3 × ULN and TBL > ULN | 2/78 (2.6) | 4/76 (5.3) | | 8/77 (10.4) | | 5/77 (6.5) | |
| AT >3 × ULN and TBL >2 × ULN | 0/78 | 1/76 (1.3) | | 3/77 (3.9) | | 2/77 (2.6) | |
| AT >5 × ULN and TBL > ULN | 0/78 | 3/76 (3.9) | | 6/77 (7.8) | | 2/77 (2.6) | |
| AT >5 × ULN and TBL >2 × ULN | 0/78 | 1/76 (1.3) | | 3/77 (3.9) | | 1/77 (1.3) | |
| AT >8 × ULN and TBL > ULN | 0/78 | 1/76 (1.3) | | 3/77 (3.9) | | 1/77 (1.3) | |
| AT >8 × ULN and TBL >2 × ULN | 0/78 | 1/76 (1.3) | | 3/77 (3.9) | | 1/77 (1.3) | |
| AT >10 × ULN and TBL > ULN | 0/78 | 1/76 (1.3) | | 3/77 (3.9) | | 1/77 (1.3) | |
| AT >10 × ULN and TBL >2 × ULN | 0/78 | 1/76 (1.3) | | 3/77 (3.9) | | 1/77 (1.3) | |
| AT >20 × ULN and TBL > ULN | 0/78 | 0/76 | | 1/77 (1.3) | | 0/77 | |
| AT >20 × ULN and TBL >2 × ULN | 0/78 | 0/76 | | 1/77 (1.3) | | 0/77 | |
| Biochemistry, Hy’s law |  |  | |  | |  | |
| ALT or AST >3 × ULN and total bilirubin >2 × ULN and ALP <2 × ULN | 0/78 | 0/76 | | 3/77 (3.9) | | 1/77 (1.3) | |
|  |  | |  | |  | |  |

Categories are based on worst postbaseline value for any given parameter, and those with multiple parameters are based on worst postbaseline value for each parameter (i.e., they need not be concurrent). The worst postbaseline value refers to the maximum postbaseline value except for ALP in the Hy’s law criteria, which refers to the minimum postbaseline value.

ALP, alkaline phosphatase; ALT, alanine aminotransferase; AST, aspartate aminotransferase; AT, aminotransferase; G, goserelin; LET, letrozole; NSAI, nonsteroidal aromatase inhibitor; PBO, placebo; RIB, ribociclib; TBL, total bilirubin; ULN, upper limit of normal.

**Table S8. Time to and duration of select AEs**

|  | **Premenopausal cohort** | | **Postmenopausal cohort** | |
| --- | --- | --- | --- | --- |
|  | **RIB + NSAI + G** | **PBO + NSAI + G** | **RIB + LET** | **PBO + LET** |
| Grade ≥2 neutropenia (based on neutrophil count) |  |  |  |  |
| Events/patients | 73/78 | 7/77 | 74/77 | 4/77 |
| Time to first occurrence, median (range), descriptive statistics, weeks | 2.1 (1.7-92.6) | 20.1 (2.1- 73.7) | 2.1 (1.9-64.1) | 17.9 (3.0-64.0) |
| Time to first occurrence, median (range), Kaplan-Meier method, weeks | 2.1 (2.1-2.3) | NR | 2.1 (2.1-2.7) | NR |
| Duration, median (range), weeks | 2.1 (0.29-30.6) | 4 (0.9-6.1) | 2.6 (0.6-24.7) | 3.1 (0.3-8.1) |
| Grade ≥3 ALT/AST elevation |  |  |  |  |
| Events/patients | 3/79 | 3/77 | 13/76 | 5/76 |
| Time to first occurrence, median (range), descriptive statistics, weeks | 53.3 (5.6-73.7) | 42.9 (4.1-53.9) | 6.1 (2.1-12.3) | 45.1 (2.1-120.1) |
| Time to first occurrence, median (range), Kaplan-Meier method, weeks | NR | NR | NR | NR |
| Duration, median (range), weeks | 0.6 (0.6-0.6) | 2.1 (0.6-3.6) | 1.6 (0.6- 3.1) | 1.1 (1.1-1.1) |
| Grade ≥2 QT prolongation (based on ECG data) |  |  |  |  |
| Events/patients | 13/79 | 2/76 | 8/76 | 0/77 |
| Time to first occurrence, median (range), descriptive statistics, weeks | 2.1 (1.7-10.1) | 4.1 (2.1-6.1) | 2.1 (2.0-128.0) | — |
| Time to first occurrence, median (range), Kaplan-Meier method, weeks | NR | NR | NR | — |
| Duration, median (range), weeks | 1.6 (0.4-2.6) | 0.8 (0.3-1.3) | 0.8 (0.3-2.3) | — |

Median duration of AE was per Kaplan-Meier method.

AE, adverse event; ALT, alanine aminotransferase; AST, aspartate aminotransferase; ECG, electrocardiogram; G, goserelin; LET, letrozole; NR, not reached; NSAI, nonsteroidal aromatase inhibitor; PBO, placebo; RIB, ribociclib.
